# *CCAT-1* promotes proliferation and inhibits apoptosis of cervical cancer cells via the Wnt signaling pathway

**DOI:** 10.18632/oncotarget.19155

**Published:** 2017-07-10

**Authors:** Jun Zhang, Yali Gao

**Affiliations:** ^1^ Department of Obstetrics and Gynecology, The Second Clinical Medical College (Shenzhen People’s Hospital), Jinan University, Shenzhen 518020, China; ^2^ Department of Ophthalmology, The Second Clinical Medical College (Shenzhen People’s Hospital), Jinan University, Shenzhen 518020, China

**Keywords:** cervical cancer, long non-coding RNA, CCAT-1, Wnt pathways, c-Myc

## Abstract

Though the long noncoding RNA colon cancer associated transcript-1 (*CCAT-1*) has been shown to be involved in tumors of other tissues, its involvement in cervical cancer is still unknown. Therefore, the aim of this study was to investigate the molecular mechanism of CCAT-1 in cervical cancer. We quantified the expression of *CCAT-1* long noncoding RNA in samples of cervical cancer tissue by real-time PCR. Effects of *CCAT-1* expression on the proliferation and apoptosis of HeLa and CaSki cells were assessed by cell-count, colony-formation, and flow cytometry assays. Binding of the c-Myc protein to the *CCAT-1* promoter was confirmed by chromatin immunoprecipitation. Finally, TOP-Flash and western blotting were used to examine the regulation of the Wnt/β-catenin pathway by *CCAT-1*. The results showed that compared with adjacent normal tissue, the expression of *CCAT-1* in cervical cancer tissue was significantly upregulated. *CCAT-1* expression was related to the stage and size of the tumor and recurrence prognosis. Then, we showed through functional assays that *CCAT-1* could promote proliferation and inhibit apoptosis of cervical cancer cells. Furthermore, chromatin immunoprecipitation showed that c-Myc protein could promote *CCAT-1* expression by binding to its promoter. Finally, fluorescent-reporter assays and western blotting showed that *CCAT-1* could activate the Wnt/β-catenin pathway. In conclusion, we showed that *CCAT-1* can be activated by the c-Myc protein and it can promote proliferation and inhibit apoptosis in cervical cancer cells by regulating the Wnt/β-catenin pathway. *CCAT-1* might serve as a good prognostic indicator and target for treatment of cervical cancer.

## INTRODUCTION

Cervical cancer is the fourth most common malignancy in women worldwide. There are appro-ximately 500,000 new cases of cervical cancer and 233,000 deaths caused by it per year [1]. Thus, cervical cancer is a major health problem worldwide. A deeper understanding of the genetic mechanisms that control the progression of the disease is imperative for enabling early clinical diagnosis and effective treatment of cervical cancer patients.

The study of long non-coding RNAs (lncRNAs) has recently gained prominence in the field of gene regulation research. LncRNAs are RNA molecules that are longer than 200 nucleotides in length and contain no protein-coding capacity [2]. Increasing evidence suggests that lncRNAs are involved in tumorigenic processes such as cell proliferation, apoptosis, differentiation, and invasion in [3, 4] in cervical cancer [5, 6], retinoblastoma [7], and oral squamous cell carcinoma [8].

Colon cancer associated transcript-1 (*CCAT-1*) is a type of lncRNA that is closely associated with colon cancer [9, 10]. However, recent studies have shown that *CCAT-1* is also involved in gastric cancer [11], hepatocellular carcinoma [12], gallbladder cancer [13], and lung cancer [14, 15]. *CCAT-1* gene is located on chromosome 8q24.21 in the vicinity of *c-Myc*, a well-known transcription factor [16–18]. Studies have shown that *c-Myc* can activate the transcription of *CCAT-1*, and the depletion of CCAT-1 RNA can reduce the effect of *c-Myc* [18, 19]. c-MYC is one of the most important oncogenes of cervical cancer [20, 21]; therefore, we propose that CCAT-1 might be involved in the progression of cervical cancer as well. However, the specific role of CCAT-1 in cervical cancer is still unclear. Further understanding of the role of *CCAT-1* in cervical cancer development may provide better therapeutic opportunities for cervical cancer patients. Thus, the present study is aimed at investigating the role of *CCAT-1* in cervical cancer.

First, the expression of *CCAT-1* in cervical cancer tissues and its relationship with clinicopathological parameters were evaluated. Then we assayed the activity of *CCAT-1* in cervical cancer cell lines. Finally, we studied the upstream and downstream regulatory factors affecting the expression of *CCAT-1* in cervical cancer cell lines.

## RESULTS

### Expression of *CCAT-1* in cervical cancer tissues and adjacent normal tissues

**Figure 1:**
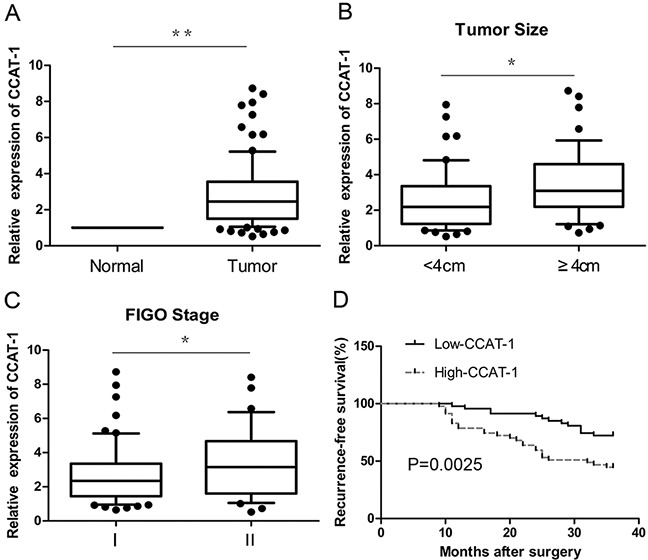
The relative expression of *CCAT-1* in cervical cancer tissues **(A)** The expression of *CCAT-1* in cervical cancer tissues (n=94) was significantly higher than that in adjacent normal tissues. **(B)** The expression of *CCAT-1* in patients with tumor size <4 cm was significantly lower than that in patients with tumor size ≥ 4 cm. **(C)** The expression of *CCAT-1* in patients of FIGO stage I was significantly lower than that in FIGO stage II. **(D)** The recurrence-free survival of low-CCAT-1 group was significantly higher than that of high-CCAT-1 group. Each assay was performed in triplicate. *P < 0.05, ^*^P < 0.01.

Expression levels of *CCAT-1* in cervical cancer tissues (n=94) and matched, adjacent, normal tissues were detected by quantitative real-time PCR (qRT-PCR). Cervical cancer tissues showed a significantly higher expression than matched, adjacent normal tissues (*P*<0.01, Figure 1A).

### Relationship between *CCAT-1* and clinicopathological parameters in cervical cancer

The results of qRT-PCR showed that the expression of *CCAT-1* was related to the FIGO (International Federation of Gynecology and Obstetrics) stage and size of the tumor (*P*<0.05, Figure 1B, 1C). However, no significant relationship was observed between *CCAT-1* levels and other parameters such as age and menopausal status of the patients, histological organization of the tissue, degree of differentiation, depth of invasion, lymphatic vascular space invasion (LVSI), and lymph node metastasis of the tumor (Table 1). Then, the median value of all 94 cervical cancer tissue samples was set as the cut-off point to separate tumors with low-level expression of *CCAT-1* (low-CCAT-1 group) from those with high-level expression of *CCAT-1* (high-CCAT-1 group). Kaplan-Meier survival analysis showed that recurrence-free survival of the low-CCAT-1 group was significantly higher than that of the high-CCAT-1 group (*P*=0.002, Figure 1D). COX univariate analysis showed that CCAT-1, FIGO stage, lymph node metastasis, and LVSI were prognostic factors for recurrence. Furthermore, COX multivariable analysis showed that only *CCAT-1* expression level, FIGO stage, and lymph node metastasis were independent prognostic factors for recurrence (Table 2).

**Table 1:** Correlation between *CCAT-1* and clinicopathological characteristics

Clinicopathologic feature	n(%)	*CCAT-1* (mean±SEM)	*P*-value
Age			0.411
≤50	52(55.3%)	2.80±0.25	
>50	42(44.7%)	3.12±0.28	
Menopause			0.712
Yes	54(57.4%)	2.88±0.25	
No	40(42.6%)	3.02±0.29	
Histology			0.918
Squamous cell cancer	69(73.4%)	2.95±0.20	
Adenocarcinoma	17(18.1%)	2.43±0.36	
Other	8(8.5%)	2.90±0.46	
Differentiation			0.925
Well to moderately	58(61.7%)	2.96±0.32	
Poorly	36(38.3%)	2.92±0.32	
Tumor size			0.022
<4cm	50(53.2%)	2.55±0.23	
≥4cm	44(46.8%)	3.41±0.29	
Depth of invasion			0.353
≤2/3	53(56.4%)	2.79±0.27	
>2/3	41(43.6%)	3.14±0.26	
Lymphatic vascular space invasion			0.390
Negative	47(50%)	2.78±0.19	
Positive	47(50%)	3.10±0.32	
Lymph node metastasis			0.122
Negative	55(58.5%)	2.72±0.22	
Positive	39(41.5%)	3.33±0.34	
FIGO stage			0.025
I	60(63.8%)	2.59±0.26	
II	34(36.2%)	3.44±0.25	

**Table 2:** Univariate and multivariate analyses for recurrence-free survival

Risk factors	Univariate analysis			Multivariate analysis		
	HR	*P*	95 % CI	HR	*P*	95 % CI
*CCAT-1* expression	1.247	0.004	1.074∼1.448	1.251	0.017	1.041∼1.504
FIGO stage, (I, II)	4.801	<0.001	2.482∼9.284	4.097	<0.001	2.111∼7.950
Lymph nodes metastasis (negative, positive)	2.280	0.011	1.209∼4.299	2.106	0.044	1.987∼4.494
LVSI (negative, positive)	2.035	0.031	1.067∼3.883	1.422	0.307	0.724∼0.279
Depth of invasion (≤2/3, >2/3)	1.565	0.163	0.835∼2.934			
Differentiation (well/moderately, poorly)	1.572	0.158	0.839∼2.948			
Tumor size	1.834	0.094	1.036∼3.396			
Age	1.006	0.673	0.978∼1.034			
Histology (squamous, adenocarcinoma)	1.246	0.580	0.572∼2.710			
Menopause (yes, no)	1.259	0.484	0.660∼2.402			

HR (hazard ratio)

### Effect of pcDNA-CCAT-1, si-CCAT-1, sh-CCAT-1, pcDNA-c-Myc, and si-c-Myc

**Figure 2:**
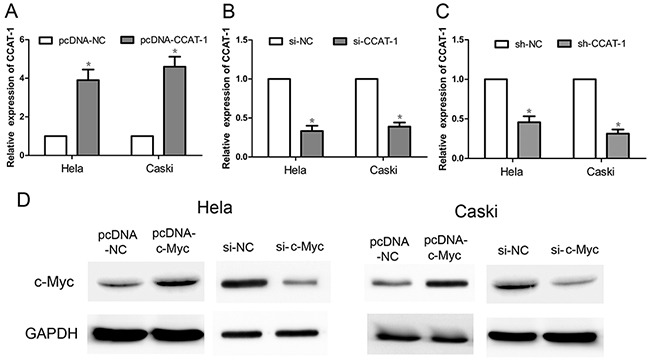
The relative expression of *CCAT-1* and *c-Myc* after transfection of cervical cancer cells **(A)** pcDNA-CCAT-1 could significantly increase the expression of *CCAT-1* in HeLa and CaSki cells. **(B, C)** si-CCAT-1 and sh-CCAT-1 could down-regulate the expression of *CCAT-1* in HeLa and CaSki cells. **(D)** pcDNA-c-Myc can significantly increase the expression of *c-Myc* protein in HeLa and CaSki cells, and si-c-Myc can significantly decrease the *c-Myc* protein expression in HeLa and CaSki cells. Each assay was performed in triplicate. *P < 0.05.

qRT-PCR results showed that the expression of *CCAT-1* was significantly increased in HeLa and CaSki cells transfected with an expression plasmid (pcDNA) carrying the *CCAT-1* gene (pcDNA-CCAT-1), compared with cells transfected with an expression plasmid carrying a scrambled negative control (pcDNA-NC; *P* <0.05, Figure 2A). In contrast, the expression of *CCAT-1* was significantly decreased in Hela and CaSki cells transfected with small-interfering CCAT-1 (si-CCAT-1) or short-hairpin CCAT-1 (sh-CCAT-1) constructs compared with their respective negative controls (si-NC and sh-NC; *P* <0.05, Figure 2B, 2C).

Western blotting showed that the expression of the c-Myc protein was significantly increased in HeLa and CaSki cells transfected with pcDNA-cMyc, compared with the control group (Figure 2D). Conversely, the expression of the c-Myc protein was significantly reduced in HeLa and CaSki cells transfected with si-cMyc compared with that in the control group (Figure 2D).

### Effect of *CCAT-1* on tumorigenic ability of cervical cancer cells

**Figure 3:**
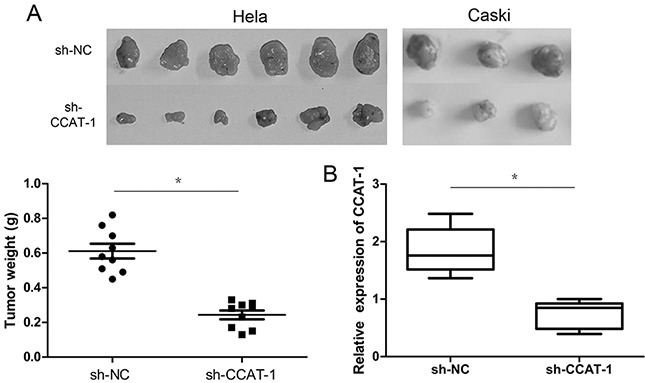
The effect of *CCAT-1* on the proliferation of cervical cancer cells in vivo **(A)** sh-CCAT-1 significantly inhibited the tumorigenic ability of HeLa and CaSki cells in vivo. **(B)** Expression levels of *CCAT-1* were detected in xenograft tumor tissues by qRT-PCR. Each assay was performed in triplicate. *P < 0.05.

Tumorigenesis assays on nude mice showed that the tumorigenic ability of HeLa and CaSki cells transfected with sh-CCAT-1 was significantly lower than cells transfected with sh-NC (*P* <0.05, Figure 3A). Furthermore, qRT-PCR confirmed that the average *CCAT-1* expression in tumors carrying sh-CCAT-1 was significantly lower than that of tumors carrying sh-NC (*P* <0.05, Figure 3B).

### Effect of *CCAT-1* on proliferation of cervical cancer cells

**Figure 4:**
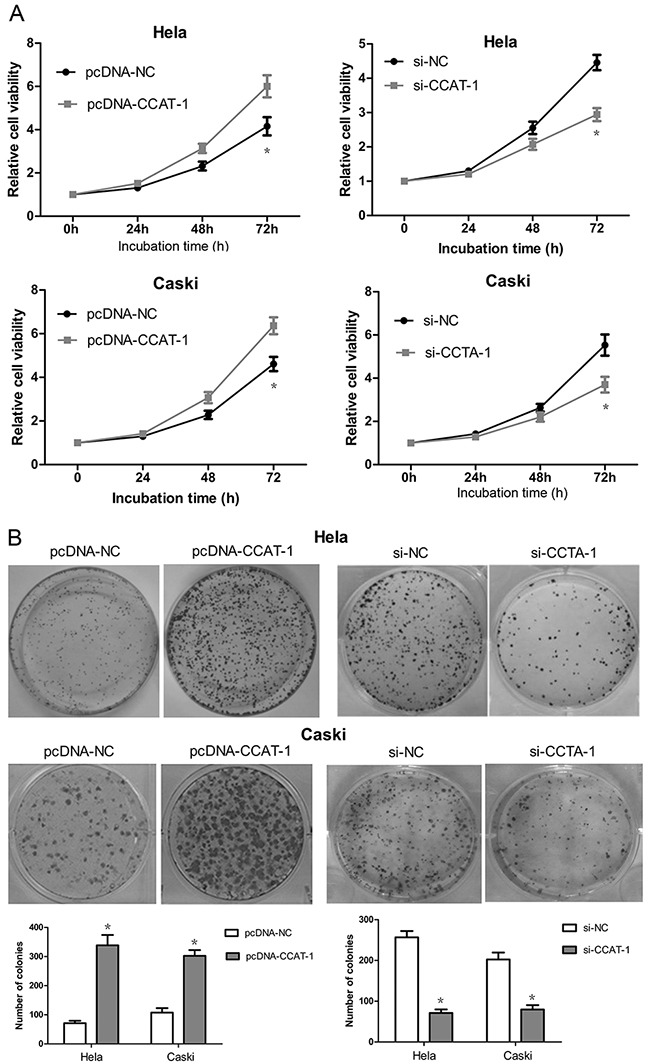
The effect of *CCAT-1* on the proliferation of cervical cancer cells in vitro **(A)** The growth curve and cell proliferative potential (CCK-8) were determined in HeLa and CaSki cells after pcDNA-CCAT-1 and si-CCAT-1 transfection compared to negative control. **(B)** Colony-formation assay was used to determine the cell proliferation ability at 48 h post-transfection. Each assay was performed in triplicate. *P < 0.05.

Cell Counting Kit-8 (CCK-8) assays showed that the proliferation of HeLa and CaSki cells transfected with pcDNA-CCAT-1 increased significantly compared with that of the negative control (*P* <0.05, Figure 4A). In contrast, the proliferation of HeLa and CaSki cells transfected with si-CCAT-1 was significantly inhibited compared to that of the control (*P* <0.05, Figure 4A).

### Effect of *CCAT-1* on colony formation by cervical cancer cells

As depicted in Figure 4B, pcDNA-CCAT-1 transfection led to a significant increase in the number of colonies formed by HeLa and CaSki cells compared with that by the negative controls. We also observed a significant decrease in the number of colonies formed by si-CCAT-1-transfected cells compared with the negative controls.

### Effect of *CCAT-1* on apoptosis of cervical cancer cells

**Figure 5:**
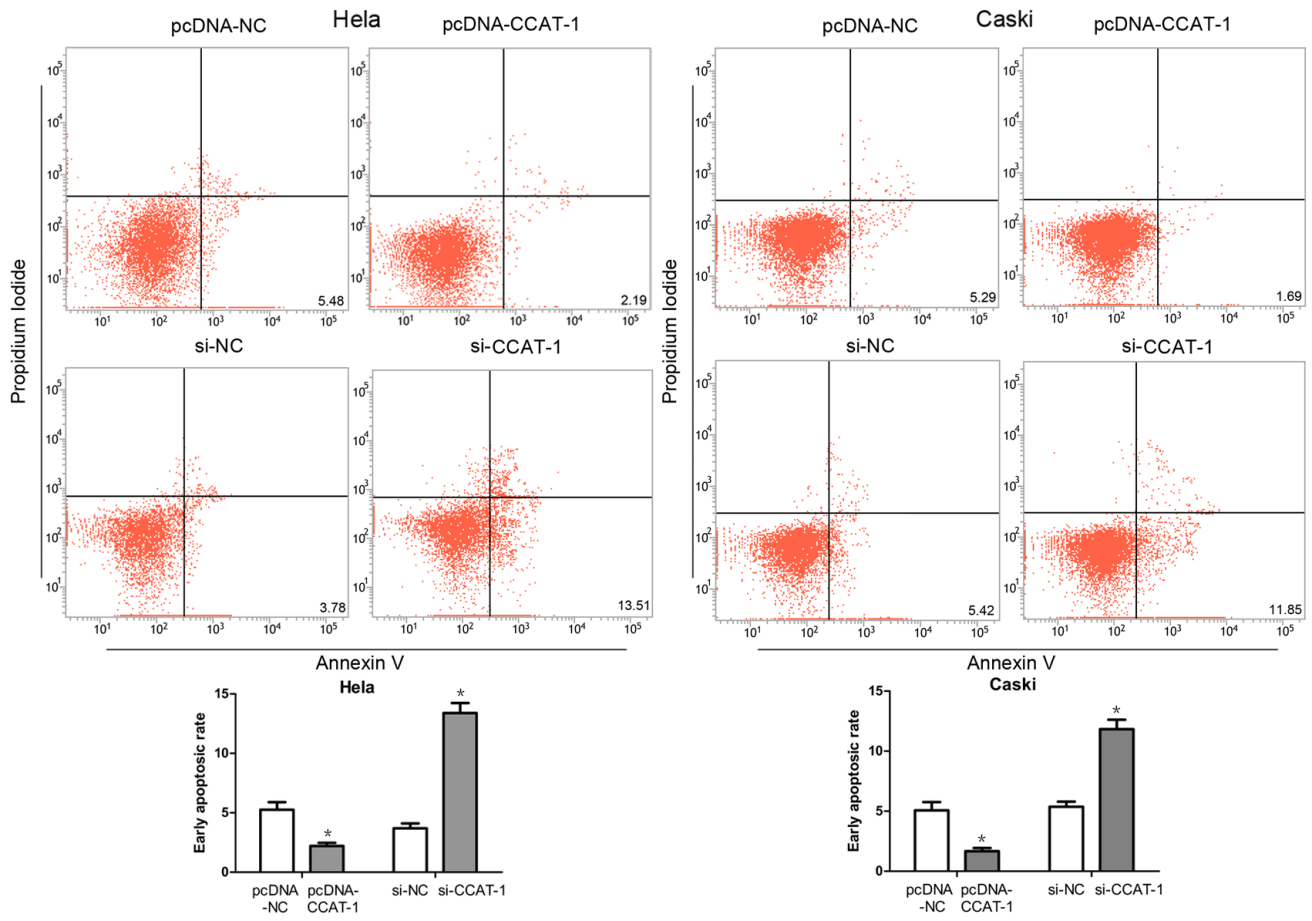
Flow cytometry was used to assess the early apoptotic rate of cervical cancer cells The proportion of early apoptotic cells in pcDNA-CCAT-1 group was significantly lower than that in pcDNA-NC group. And the percentage of early apoptotic cells in HeLa and CaSki cells transfected with si-CCAT-1 was significantly higher than that in control cells. Each assay was performed in triplicate. *P < 0.05.

Flow cytometry showed that the percentage of early apoptotic cells among HeLa and CaSki cells transfected with pcDNA-CCAT-1 was significantly lower than that among control cells (*P* <0.05, Fig. 5). And compared with the control group, the percentage of early apoptotic cells in HeLa and CaSki cells transfected with si-CCAT-1 was significantly higher (*P* <0.05, Figure 5).

### Effect of *c-Myc* on the expression of *CCAT-1* in cervical cancer cells

**Figure 6:**
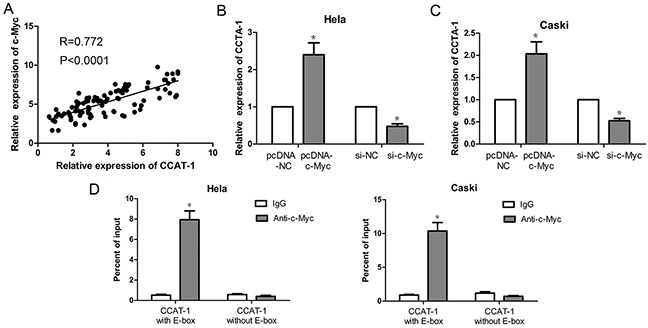
Expression of *CCAT-1* was up-regulated by *c-Myc* protein **(A)** There was a positive correlation between *CCAT-1* and *c-Myc* mRNA expression in cervical cancer tissues (n=94). **(B, C)** Up-regulation of the expression of *c-Myc* protein in HeLa and CaSki cells could promote the transcription of *CCAT-1*. And down-regulated the expression of *c-Myc* protein in HeLa and CaSki cells could inhibit the transcription of *CCAT-1*. **(D)** ChIP assay was used to detect the binding of *c-Myc* protein to E-box of *CCAT-1* promoter. Each assay was performed in triplicate. *P < 0.05.

First, our results showed that the expression of *CCAT-1* was positively correlated with the expression of *c-Myc* in 94 samples of cervical cancer tissue (*P*<0.0001, Figure 6A). Then, in HeLa and CaSki cells the expression of *CCAT-1* was significantly increased upon pcDNA-cMyc transfection and significantly decreased upon si-cMyc transfection, compared with that in their controls (*P* <0.05, Figure 6B, 6C).

Furthermore, we examined the binding of c-Myc to the E-box of the *CCAT-1* promoter region using chromatin immunoprecipitation assay. For *CCAT-1* promoter containing a wild-type E-box, we found that the amount of c-Myc immunoprecipitates was significantly higher than that of lgG immunoprecipitates (*P* <0.05, Figure 6D). However, for the *CCAT-1* promoter with the E-box absent, there was no difference in binding between c-Myc and lgG as revealed by the lack of statistically significant difference in the amount of c-Myc and IgG immunoprecipitates (Figure 6D).

### Effect of *CCAT-1* on the activity of Wnt pathway

**Figure 7:**
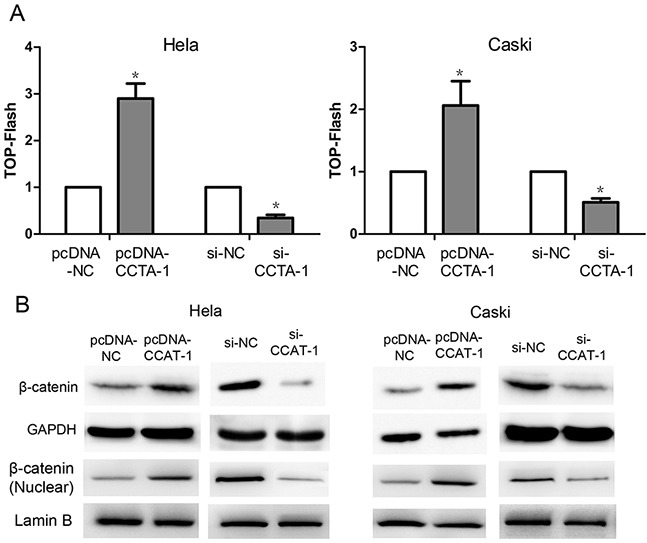
The regulation of Wnt pathway by *CCAT-*1 **(A)** TOP-Flash assays showed that *CCAT-1* might up-regulate the Wnt pathway activity and down-regulation of *CCAT-1* might significantly decrease the Wnt pathway activity of HeLa and CaSki cells. **(B)** Western blot showed that up-regulation of *CCAT-1* expression in HeLa and CaSki cells resulted in a significant increase of β-catenin protein level, while down-regulation of *CCAT-1* in HeLa and CaSki cells resulted in a significant decrease of β-catenin protein level. Each assay was performed in triplicate. *P < 0.05.

The activity of luciferase was found to be significantly higher in HeLa and CaSki cells transfected with pcDNA-CCAT-1 construct compared with cells transfected with the control pcDNA-NC construct by TOP Flash assays. Conversely, the activity of luciferase was significantly lower in HeLa and CaSki cells transfected with si-CCAT-1 compared with that in control cells (*P* <0.05, Figure 7A). In addition, western blotting showed that β-catenin protein levels were significantly increased in the pcDNA-CCAT-1 transfected group compared with the pcDNA-NC transfected group. Conversely, the level of β-catenin in HeLa and CaSki cells transfected with si-CCAT-1 was significantly lower than that in the controls (P <0.05, Figure 7B).

**Figure 8:**
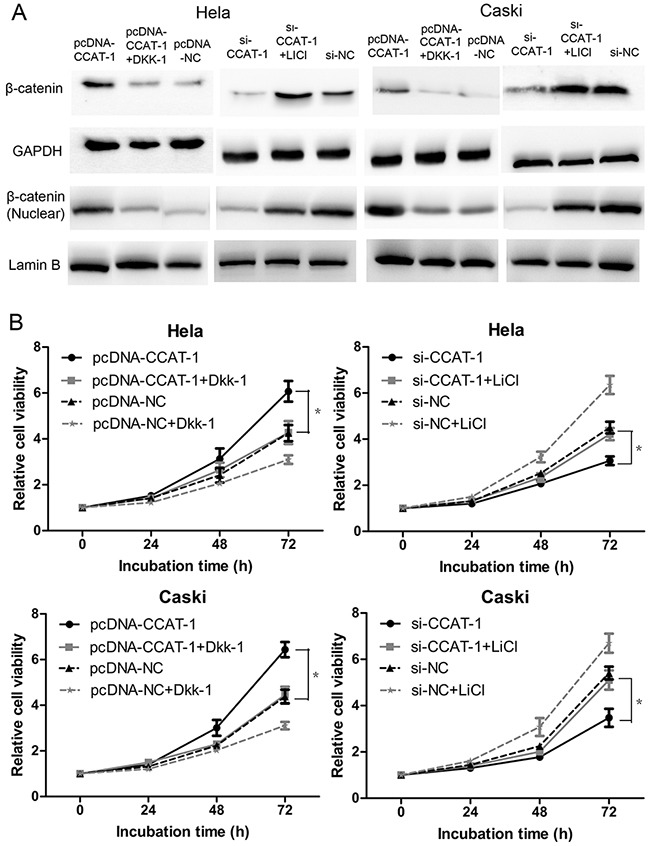
*CCAT-1* achieved its biological functions via regulating Wnt pathway **(A)** Wnt pathway antagonist Dkk-1 and activator LiCl just reversed the regulatory effect of pcDNA-CCAT-1 and si-CCAT-1 on Wnt pathway. **(B)** The *CCAT-1* biological function was impaired after reversing the regulatory effect of *CCAT-1* on Wnt pathway. Each assay was performed in triplicate. *P < 0.05.

Finally, we used the Wnt/β-catenin pathway anta-gonist Dkk-1 and the Wnt/β-catenin pathway acti-vator LiCl to reverse the effects of pcDNA-CCAT-1 and si-CCAT-1 in HeLa and CaSki cells (Figure 8A). CCK-8 assays showed that the proliferation of HeLa and CaSki transfected with pcDNA-CCAT-1 and Dkk-1 was significantly lower than cells transfected only with pcDNA-CCAT-1. Conversely, the proliferation of HeLa and CaSki si-CCAT-1 transfected cells was significantly enhanced when they were treated with LiCl compared with untreated cells (*P* <0.05, Figure 8B).

## DISCUSSION

The study of lncRNAs has gained prominence in the field of gene regulation in recent years. Accumulated evidence from recently published studies suggests that aberrant lncRNA expression may be a crucial component of tumorigenesis in cervical cancer [22–24]. CCAT-1, a newly discovered lncRNA, is involved in the development of tumors in many different tissues [9–16]. However, its role in cervical cancer is unknown.

In this study, we first discovered that *CCAT-1* was overexpressed in cervical cancer tissue compared with that in normal, adjacent tissue. Then we analyzed the relationship between *CCAT-1* expression and clinicopathological characteristics of the patients. We found that *CCAT-1* expression was associated with the FIGO stage and size of the tumor. *CCAT-1* shows a similar overexpression in other cancers [9–16]. Since FIGO stage and tumor size are key factors in the prognosis of cervical cancer, we hypothesized that *CCAT-1* expression levels too might show a direct correlation with prognosis. Our data confirmed that *CCAT-1* expression served as an independent prognostic factor for cervical cancer. Our results indicate that *CCAT-1* expression can be employed as a potential tool for preoperative identification and postoperative follow-up of patients at high risk for recurrent cervical cancer.

Since we observed that *CCAT-1* expression was associated with tumor size, we speculated that it has a role in promoting the proliferation of cervical cancer cells. Therefore, we conducted CCK-8 and flow cytometry assays on cells that were made to overexpress *CCAT-1*. We found that *CCAT-1* overexpression promoted proliferation and inhibited apoptosis in cervical cancer cell lines, HeLa and CaSki. Conversely, silencing the expression of *CCAT-1* by RNA interference inhibited proliferation and promoted apoptosis in cervical cancer cell lines. These results confirmed that CCAT-1 functions by promoting cervical cancer cell growth. We simultaneously conducted tumor formation assays on nude mice injected with HeLa and CaSki cells carrying recombinant *CCAT-1* constructs and confirmed that silencing of *CCAT-1* expression significantly inhibited the tumorigenicity of cervical cancer cells. Although these results may be corroborated by manipulating CCAT1 expression in tumors of other tissues, they did demonstrate that *CCAT-1* promoted tumor growth and functioned as an oncogene in cervical cancer in our study.

Previous studies have shown that *c-Myc* can activate *CCAT-1* expression in colon and gastric cancers, and human bronchial epithelial cells [10, 11, 14, 15]. Additionally, the *c-Myc* gene has also been confirmed to promote the tumorigenicity of cervical cancer cells [20, 21]. However, whether *c-Myc* can activate *CCAT-1* expression in cervical cancer cells remains unknown. We first analyzed the relationship between the expression of *CCAT-1* and *c-Myc* in samples of cancerous tissue and found that the two are positively correlated, suggesting that there may be a regulatory relationship between *CCAT-1* and *c-Myc*. Subsequently, we found that *CCAT-1* expression was significantly upregulated upon overexpressing *c-Myc* in cervical cancer cells. In contrast, silencing *c-Myc* expression significantly inhibited *CCAT-1* expression in cervical cancer cells. These results suggested that *c-Myc* activity upregulates the expression of *CCAT-1* in cervical cancer cells. Furthermore, to determine whether c-Myc could directly bind to sites within the *CCAT-1* promoter, chromatin immunoprecipitation assays were performed. We found that c-Myc could bind to the wild-type E-box within the *CCAT-1* promoter, but could not bind to the *CCAT-1* promoter when the E-box was absent. Together, these results systematically revealed a mechanism for expression of *CCAT-1* in cervical cancer cells: *c-Myc* promotes the expression of *CCAT-1* by binding to the E-box of its promoter, leading to the proliferation of cervical cancer cells.

The Wnt/β-catenin pathway regulates several cellular processes such as proliferation, invasion, and differentiation [25]. Previous studies have shown that Wnt/β-catenin pathway activation can promote cell proliferation and inhibit cell apoptosis in cervical cancer [26, 27]. Therefore, we also aimed to explore the regulatory relationship between *CCAT-1* and the Wnt/β-catenin pathway. We used TOP-Flash reporter assays to first show that downregulation of *CCAT-1* can significantly inhibit Wnt/β-catenin pathway activity in cervical cancer cells. Furthermore, we found that upregulating the expression of *CCAT-1* could significantly activate the Wnt/β-catenin pathway in cervical cancer cells. Western blotting confirmed that upregulation of *CCAT-1* significantly increased the expression of the β-catenin protein, while downregulation of *CCAT-1* expression resulted in a significant reduction in the expression of the β-catenin protein. It has been demonstrated in previous studies that LiCl activates the Wnt/β-catenin pathway by inhibiting GSK3β and stabilizing β-catenin, while DKK-1 can inhibit the Wnt/β-catenin pathway by binding to LRP6 [28, 29]. Therefore, we used DKK-1 as an antagonist and LiCl as an agonist to reverse the effects of *CCAT-1* manipulation on the Wnt/β-catenin pathway in cervical cancer cells. The functional assays revealed that the biological function of CCAT-1 was significantly weakened in cervical cancer cells after the reversal. These results suggested that the Wnt/β-catenin pathway is an important downstream target of *CCAT-1* for promoting proliferation and inhibiting apoptosis in cervical cancer cells.

Altogether, we propose the following model for the proliferation of cervical cancer cells: overexpression of *c-Myc* → enhancement of *CCAT-1* gene transcription → activation of Wnt/β-catenin pathway → enhanced cell proliferation. This study helps us to better understand the specific role of CCAT-1 in malignant growth of cervical cancer cells.

In summary, our study indicated that *CCAT-1* overexpression was related to tumor size and prognosis in cervical cancer. The expression of *CCAT-1* might be caused by the binding of the c-Myc protein to the promoter of *CCAT-1*. *CCAT-1* might promote proliferation and inhibit apoptosis of cervical cancer cells by activating the Wnt/β-catenin pathway. In conclusion, the study and manipulation of *CCAT-1* may prove to be useful for prognosis and treatment of cervical cancer.

## MATERIALS AND METHODS

### Clinical samples

A total of 94 paired cervical cancer tissues and adjacent normal tissues were obtained from the cervical cancer patients with surgical treatment between January 2011 and May 2012. Freshly specimens were immediately frozen in liquid nitrogen and stored at −80°C until used. All patients did not receive any preoperative tumor-related treatment, and pathology was confirmed as cervical cancer by two pathologists. Written informed consent was obtained from each patient, and the present study was approved by the ethics committee of University. The clinical and pathological data of patients was shown in Table 1.

### Cell culture conditions

Human cervical cancer cell lines (HeLa, CaSki) were obtained from the Cell Bank of the Chinese Academy of Sciences. Cells were cultured in DMEM (Thermo Scientific, Belmont, Massachusetts, US) containing 10% fetal bovine serum (Gibco, Gaithersburg, USA) at 37°C in a humidified chamber supplemented with 5% CO_2_.

### Establish the stable shRNA-CCAT-1 cervical cancer cell lines by lentivirus

The lentiviral vectors with shRNA-CCAT-1 were constructed by the GenePharma Company (Shanghai, China). Briefly, the oligonucleotide encoding shRNA-CCAT-1 and negative control were annealed and inserted into the pCMV vector (GenePharma). The recombinational vectors and the packaging vectors were co-transfected into 293T cells (Invitrogen, Carlsbad, USA) with Lipofectamine2000 (Invitrogen, USA). The culture supernatants were collected at 48 hours after transfection, concentrated. The cells were divided into two groups according our objectives: infection of control virus (sh-NC); infection of shRNA-CCAT-1 virus (sh-CCAT-1). The shRNA-CCAT1 sequences used showed in Supplementary Table 1.

### Transient transfection

pcDNA-CCAT-1, si-CCAT-1, pcDNA-c-Myc, si-c-Myc and their negative controls were purchased from GenePharma Company (Shanghai, China). Transient transfection was performed using the Lipofectamine 2000 (Invitrogen, USA) following the manufacturer’s instructions. Forty-eight hours after transfection, the cells were collected for further experiments. siRNA sequences were listed in Supplementary Table 1.

### RNA extraction and quantitative real-time PCR (qRT-PCR)

Total RNAs from tissues and cells were extracted using TRIzol (TaKaRa, Otsu, Japan). After reverse transcription reaction with Prime-ScriptTM one step RT-PCR kit (TaKaRa, Otsu, Japan), qPCR was conducted with SYBR Premix Ex Taq (TaKaRa, Otsu, Japan) on CFX96™ Real-Time PCR Detection System supplied with analytical software (Bio-Rad, USA). GAPDH were used as endogenous control and the relative expression of RNAs was calculated using the 2^−ΔΔCt^ method. The primers used in this study were synthesized by GenePharma Company (Shanghai, China) and the sequences were shown in Supplementary Table 2.

### Cell proliferation assay

Cell viability was determined using the CCK8 assay according to the manufacturer’s protocol. Briefly, Cells were plated in 96-well plates in triplicate at 5×10^3^ per well in a final volume of 90 μl. Then 10 μl CCK8 solutions was added to each well and incubated for 2h at 37°C and 5% CO_2_ at each time point (0, 24, 48, 72h). After incubating, the absorbance at 450 nm was measured by an SpectraMax M5 (Molecular Devise, California, USA).

### Apoptosis assay

Apoptotic fractions cells were analysed by Annexin-V-FLUOS Kit (BD Pharminogen, USA) according to the manufacturer’s instructions. Annexin V and PI was added 48h after transfection and apoptotic cells were identified with a flow cytometer (FACSCalibur, BD Biosciences). The relative ratio of early apoptotic cells were counted for further comparisons.

### Colony-formation assay

Cells were plated at 500 per well into 6-well plates in DMEM supplemented with 10% FBS. Dishes were incubated at 37°C in a humidified atmosphere containing 5% CO2. After 7 days, cells were washed twice with PBS, fixed with formaldehyde for 30 min, stained with crystal violet (Beyotime; Shanghai, China) for 10 min, washed with ddH_2_O three times and then photographed with a digital camera.

### Xenograft tumor model

HeLa and CaSki cells (2×10^7^cells) that stably transfected with pCMV/shRNA-CCAT-1 or pCMV/shRNA-NC were re-suspended in 150 μl PBS and subcutaneously injected into the flank tissues of 5-week-old BALB/c-nu female mice, respectively. 21 days after inoculation, the animals were sacrificed, the xenografts were isolated and the weight (gram) of the xenografts was determined. The protocols for animal experiment study were approved by the Institutional Committee for Animal Research in University.

### Western blot analysis

Cells were lysed at 4°C for 30 min using RIPA buffer (Thermo Scientific, Massachusetts, USA) that contained the protease inhibitor cocktail (Roche, Basel, Switzerland). Nuclear protein extracts were prepared using the Nuclear Extraction Kit (Sangon Biotechology, Shanghai, China) according to the manufacturer’s instructions. Protein lysates were separated by 10% SDS-polyacrylamide gel electrophoresis (SDS-PAGE) and transferred onto PVDF membrane (Roche, Basel, Switzerland). The membrane was blocked in 5% nonfat milk for 1 h at room temperature and subsequently incubated with specific antibody against β-catenin (1:1000, Cell Signaling Technology, Danvers, USA) and c-Mcy (1:1000, Cell Signaling Technology, Danvers, USA) overnight at 4°C. Afterwards, the membrane was washed and incubated with HRP Goat-anti-Rabbit (1:2000; Santa Cruz Biotechnology; Dallas, USA) for 1 h. Protein detection was performed using an ECL chemiluminescence reagent (Thermo Fisher Scientific, MA, USA). Lamin B (1:200, Santa Cruz Biotechnology) served as a nuclear protein internal control and GAPDH (1:1000, Cell Signaling Technology, Danvers, USA) served as a total protein internal control.

### Chromatin immunoprecipitation assay

Chromatin immunoprecipitation (ChIP) assays were performed according to the EZ ChIP Chromatin Immunoprecipitation Kit (Millipore, Bedford, MA, USA). Briefly, HeLa and CaSki cells were treated with formaldehyde and incubated for 10 min to generate DNA-protein cross-links. Cell lysates were then sonicated to generate chromatin fragments of 200–300 bp and immunoprecipitated with antibodies against *c-Myc* (Santa Cruz Biotechnology, Santa Cruz, CA, USA) or IgG as control. The antibody-bound complex was precipitated by Protein A-Sepharose beads. The DNA fragments in the immunoprecipitated complex were released by reversing the cross-linking at 65°C for 5 hour and purified DNA was analyzed by qRT-PCR with SYBR-Green incorporation (Applied Biosystems, Foster City, CA, USA). The ΔCt was calculated as ΔCt (normalized ChIP) = [Ct (ChIP) – Ct (Input)]. The % input was shown as 2^[−ΔCt(normalized ChIP)]^. The primers specific for the *CCAT1* promoter containing E-box and non-E-box were shown in Supplementary Table 2.

### TOP Flash/FOP-Flash reporter assay

The β-catenin reporter plasmid (TOP-flash) and its mutant control (FOP-flash) were constructed by Millipore Corporation (Massachusetts, USA). Cells were serum-starved overnight and co-transfected with 0.2 μg TOP flash or FOP flash expression plasmids and 0.1 μg pRL-TK (Renilla TK-luciferase vector; Promega, Madison, USA) using Lipofectamine 2000. The activities of both firefly and Renilla luciferase reporters were determined at 48 hours after transfection using a Dual Luciferase Assay Kit (Promega, Madison, WI, USA) according to the manufacturer’s instructions. The TOP-Flash reporter activity is presented as the relative ratio of firefly luciferase activity to Renilla luciferase activity, and the TOP/FOP ratio was used as a measure of β-catenin-driven transcription.

### Treatment with wnt activators and wnt antagonists

The following regulators of the Wnt signalling pathway were used: Wnt activators LiCl (Sigma-Aldrich, MO, USA) and Wnt antagonist Dickkopf-1 (Dkk1, PeproTech, New Jersey, USA). At 24 hours after transfection, the transfected cells were treated for 48 hours with LiCl (25mmol/L) or Dkk1 (120ng/ml). Following that, cells were collected for further experiments.

### Statistical analysis

SPSS 11.0 and GraphPad Prism software was used for statistical analyses. Values were expressed as mean ± standard error. Student’s t-test was used to analyze the differences between two groups and *P* < 0.05 was considered statistically significant. All experiments were performed at least three times and all samples analyzed in triplicate.

## SUPPLEMENTARY MATERIALS TABLES



## Abbreviations

lncRNA: long noncoding RNA
CCAT-1: colon cancer associated transcript 1
ChIP: chromatin immunoprecipitation
CCK-8: Cell Counting Kit-8
LVSI: lymphatic vascular space invasion
qRT-PCR: quantitative real-time polymerase chain reaction
