# Supplementary material for: CCAT-1 promotes proliferation and inhibits apoptosis of cervical cancer cells via the Wnt signaling pathway
Source: Oncotarget. 2017 Jul 10;8(40):68059–70. doi: 10.18632/oncotarget.19155 (PMC5620236; doi:10.18632/oncotarget.19155)
Supplement: Supplementary file 1 [file oncotarget-08-68059-s001.pdf]

CCAT-1 promotes proliferation and inhibits apoptosis of cervical cancer cells via the Wnt signaling pathway

SUPPLEMENTARY MATERIALS

Supplementary Table 1: Sequences of siRNA and shRNA

Name	Sequence
si-CCAT-1	5′-CCATTCCATTCATTTCTCTTTCCTA-3′
si-c-Myc	5′-GGUGAUCCAGACUCUGACCUU-3′
shRNA-CCAT1	5′-CACCCCATTCCATTCATTTCTCTTTCCTATTCAAGAGATAGGAAAGAGAAATGAATGGAATGGTTTTTTG-3′ (forward)<br>5′-GATCCAAAAAACCATTCCATTCATTTCTCTTTCCTATCTCTTGAATAGGAAAGAGAAATGAATGGAATGG-3′ (reverse)

Supplementary Table 2: PCR primers

Gene	Forward primer (5’-3’)	Reverse primer(5’-3’)
CCAT-1	5′- TTTATGCTTGAGCCTTGA-3′	5’- CTTGCCTGAAATACTTGC-3’
GAPDH	5′- GCATCCTGGGCTACACTG -3′	5′- TGGTCGTTGAGGGCAAT-3′
c-Myc	5′- GGGCTTTATCTAACTCGCTGTA-3′	5′- GCTATGGGCAAAGTTTCGTG-3′
CCAT1-E-box	5′-AGTCACTGGTGTTCTTGC-3′	5′-GGTATGCGTAGGTGATAGT-3′
CCAT1-non-E-box	5′-TCCCACCAACACCTTTAG-3′	5′-CCAAGAAGTTCTCCTCCA-3′
